# Serum Copper-to-Zinc and Copper-to-Selenium Ratios in Inflammatory Bowel Disease: Possible Associations with Clinical Disease Activity and Endoscopic Healing in a Cross-Sectional Study with Prospective Enrollment

**DOI:** 10.3390/nu18213593

**Published:** 2026-10-29

**Authors:** Chao-Feng Chang, Yi-Chiao Cheng, Chang-Hung Liao, Puo-Hsien Le, Tien-Yu Huang

**Affiliations:** 1 Division of Gastroenterology, Department of Internal Medicine, Tri-Service General Hospital, National Defense Medical University, Taipei 114, Taiwan; taiwanvincent777@gmail.com (C.-F.C.); joseph50333@gmail.com (C.-H.L.); 2 Taiwan Association for the Study of Intestinal Diseases, Taoyuan 333, Taiwan; puohsien@gmail.com; 3 Taiwan Society of Inflammatory Bowel Disease, Taipei 100, Taiwan; ndmcjoe@gmail.com; 4 Division of Colon and Rectal Surgery, Department of Surgery, Tri-Service General Hospital, National Defense Medical University, Taipei 114, Taiwan; 5 Department of Gastroenterology and Hepatology, Chang Gung Memorial Hospital, Linkou Branch, Taoyuan 333, Taiwan

**Keywords:** micronutrients, trace element homeostasis, Crohn’s disease, ulcerative colitis, nutritional assessment

## Abstract

Background/Objectives: The associations of serum copper-to-zinc (Cu/Zn) and copper-to-selenium (Cu/Se) ratios with clinical disease activity and endoscopic healing in inflammatory bowel disease (IBD) remain incompletely characterized. Although trace element imbalances have been linked to intestinal inflammation, the clinical utility of composite trace element ratios remains unclear. We investigated whether serum copper-to-zinc (Cu/Zn) and copper-to-selenium (Cu/Se) ratios reflect clinical disease activity and mucosal status in IBD. Methods: This cross-sectional study with prospective enrollment recruited patients with IBD and non-IBD controls between July 2023 and October 2024. Serum Cu, Zn, and Se concentrations were measured to calculate Cu/Zn and Cu/Se ratios. Clinical disease activity was assessed using the Crohn’s Disease Activity Index (CDAI) and partial Mayo score. Endoscopic healing was defined as the absence of ulcers or erosions on endoscopy within 3 months of enrollment. Results: Among 190 participants, 62 had Crohn’s disease (CD), 69 had ulcerative colitis (UC), and 59 were non-IBD controls. Cu/Zn and Cu/Se ratios differed across controls and clinical activity categories (both overall *p* = 0.001), with the highest mean values in patients with moderate-to-severe activity. In the overall IBD cohort, AUC values for distinguishing moderate-to-severe activity from remission or mild activity were 0.749 and 0.736 for Cu/Zn and Cu/Se, respectively. Both ratios remained higher in patients with moderate-to-severe activity than in controls after adjustment for age, sex, and BMI (both *p* < 0.001). In CD, both ratios correlated positively with CDAI (Cu/Zn: rho = 0.500; Cu/Se: rho = 0.498; both *p* < 0.001). Cu/Zn and Cu/Se ratios differed across controls and patients with and without endoscopic healing (overall *p* < 0.001 and *p* = 0.003, respectively); however, the Cu/Zn difference between the two patient groups was not statistically significant. Conclusions: Serum Cu/Zn and Cu/Se ratios were associated with clinical disease activity, but their utility for assessing endoscopic healing and their incremental value beyond established biomarkers remain uncertain. Longitudinal validation is needed before routine clinical use can be supported.

## 1. Introduction

Inflammatory bowel disease (IBD), encompassing ulcerative colitis (UC) and Crohn’s disease (CD), is characterized by chronic gastrointestinal inflammation driven by complex interactions among genetic susceptibility, epigenetics, gut microbiota dysbiosis, environmental exposures, and immune dysregulation. An estimated 6.8 million individuals were affected worldwide in 2017, and the incidence of IBD continues to rise in newly industrialized regions such as Asia [1,2,3].

Patients with IBD frequently develop trace element deficiencies due to impaired intestinal absorption, increased mucosal losses, chronic inflammation, and dietary restrictions [4]. These disturbances may occur even during clinical remission and contribute to a vicious cycle of persistent inflammation, impaired mucosal healing, and malnutrition [5,6]. Because trace elements play critical roles in immune regulation, oxidative stress responses, inflammatory response modulation, and maintenance of intestinal barrier function and integrity, their monitoring and correction are essential components of comprehensive IBD care [4,5,6]. However, routine evaluation remains underutilized in clinical practice.

Disease severity in IBD is closely associated with nutritional status and trace element homeostasis [4,7,8]. Deficiencies of zinc, selenium, and iron have been associated with increased disease activity and adverse clinical outcomes in IBD [9,10,11,12]. Among trace elements, copper, zinc, and selenium appear particularly relevant to IBD pathophysiology. Copper behaves as a pro-inflammatory acute-phase reactant, whereas zinc and selenium exert anti-inflammatory and immunomodulatory effects [5,7,9,10,11,13]. Consequently, composite trace element ratios such as copper-to-zinc (Cu/Zn) and copper-to-selenium (Cu/Se) may better reflect the balance between inflammatory and protective pathways than individual trace element measurements alone [14,15]. The Cu/Se ratio has also been investigated as a candidate biochemical marker in recurrent aphthous stomatitis, in which elevated ratios were observed alongside altered oxidative stress markers [16]. However, its relationship with clinical disease activity and endoscopic healing in IBD remains insufficiently characterized. These observations provide a rationale for evaluating both ratios in patients with IBD.

Most previous studies have focused on individual trace elements and Western populations, whereas data regarding composite trace element ratios in Asian patients with IBD remain limited. Therefore, in this study, we aimed to evaluate the associations of serum Cu/Zn and Cu/Se ratios with clinical disease activity and endoscopic healing in a Taiwanese IBD cohort.

## 2. Materials and Methods

### 2.1. Study Design and Patient Population

This cross-sectional study was conducted at the outpatient department of Tri-Service General Hospital in Taiwan between July 2023 and October 2024. We recruited adult patients (aged 20–70 years) diagnosed with IBD and a control group of individuals without IBD. The diagnosis of IBD was established by experienced gastroenterologists based on a combination of clinical presentation and endoscopic, radiological, and histopathological findings, according to established European Crohn’s and Colitis Organization (ECCO) diagnostic criteria [17,18]. The control group consisted of individuals undergoing routine health examinations who had no history of IBD, chronic gastrointestinal disorders, autoimmune diseases, or active infections.

The exclusion criteria for patients with IBD were as follows: (1) diagnosis of short bowel syndrome, (2) current receipt of enteral or intravenous nutrition, and (3) use of trace element supplements within 3 months prior to study enrollment. Individuals with major chronic diseases, a history of significant bowel surgery, overt gastrointestinal diseases, use of trace element supplement within 3 months prior to study enrollment, or active infectious diseases were excluded from the non-IBD control cohort.

Written informed consent was obtained from all participants. The study protocol was approved by the Institutional Review Board of Tri-Service General Hospital (TSGHIRB No. A202305042, Approval Date: 10 May 2023 and A202405089, Approval Date: 17 June 2024).

### 2.2. Clinical and Endoscopic Assessment

Clinical disease activity was assessed at the time of enrollment. For patients with CD, disease activity was classified according to the Crohn’s Disease Activity Index (CDAI) [19] as remission (<150), mild (150–219), moderate (220–450), or severe (>450). For patients with UC, disease activity was categorized using the partial Mayo score [20] according to established thresholds as remission (0–1), mild disease (2–4), moderate disease (5–6), or severe (7–9).

Endoscopic findings were obtained from colonoscopy, ileocolonoscopy, or deep enteroscopy performed within 3 months of enrollment and were retrospectively reviewed. Endoscopic healing was defined as the complete absence of mucosal ulcers or erosions [21].

### 2.3. Laboratory Measurements

Blood samples were collected from all participants at the Tri-Service General Hospital for biochemical analysis. Blood samples for serum trace element measurements were collected at the enrollment visit. Serum copper, zinc, and selenium concentrations were measured using standardized trace element assays at the hospital Trace Element Center, and Cu/Zn and Cu/Se ratios were calculated by dividing the serum Cu concentration by the corresponding Zn and Se concentrations, respectively, with all concentrations expressed in μg/L. These laboratory results were subsequently evaluated for correlations with the patients’ clinical and endoscopic status.

### 2.4. Statistical Analysis

Statistical analyses were performed using IBM SPSS Statistics version 20 (IBM Corp., Somers, NY, USA). Continuous variables are presented as mean ± standard deviation or median (interquartile range), as appropriate, whereas categorical variables are presented as frequencies and percentages. Comparisons among groups were performed using the Student *t*-test, Mann–Whitney U test, chi-square test, or Kruskal–Wallis test, as appropriate. Spearman correlation analysis was used to evaluate the associations between trace element ratios and disease activity indices. Receiver operating characteristic curve analysis was performed to assess the ability of the Cu/Zn and Cu/Se ratios to discriminate active disease. Statistical significance was defined as a two-sided *p*-value < 0.05. Multivariable linear regression analyses were performed to estimate differences in serum trace element concentrations and their ratios between groups, adjusting for age, sex, and BMI. Healthy controls served as the reference group for comparisons by disease type and disease activity category. Results are reported as unstandardized regression coefficients (B) with 95% confidence intervals.

## 3. Results

### 3.1. Study Population Characteristics

A total of 190 individuals were enrolled, including 62 patients with CD, 69 with UC, and 59 non-IBD controls. Baseline characteristics are summarized in Table 1. Age and sex distributions were comparable among groups. Patients with IBD had a lower body mass index than controls, particularly those with CD (*p* = 0.011). Previous bowel resection was more common in CD than in UC (32.3% vs. 4.3%, *p* < 0.001). Among patients with IBD, 77.4% of those with CD and 50.7% of those with UC were in clinical remission. The most common disease location in CD was ileocolonic involvement (45.2%), while extensive colitis was the predominant UC phenotype (55.1%).

### 3.2. Serum Microelement Profiles

Serum trace element concentrations differed across healthy controls, patients with CD, and patients with UC (Additional File S1: Table S1). Overall group differences were significant for Mg, Ca, Mn, Zn, Se, Mo, and Cu (*p* = 0.013, 0.015, 0.017, 0.001, 0.009, 0.002, and 0.047, respectively). Mean Mg, Ca, Mn, Zn, and Se concentrations were numerically lower in both patient groups than in controls, whereas mean Cu and Mo concentrations were higher. The overall comparison for Fe yielded *p* = 0.050. Direct comparisons between CD and UC showed lower Mg, Ca, and Zn concentrations in CD (*p* = 0.036, 0.004, and 0.001, respectively).

### 3.3. Correlation with Disease Activity

Comparisons across healthy controls and patients with IBD grouped by clinical disease activity showed significant overall differences in serum Cu, Se, and Mo concentrations (*p* = 0.020, 0.004, and 0.016, respectively; Additional File S1: Table S2). The moderate-to-severe activity group had the highest mean Cu concentration and the lowest mean Se and Mo concentrations. The overall difference in Zn concentrations did not reach statistical significance (*p* = 0.055). Comparisons restricted to healthy controls and the CD activity groups showed significant overall differences in Mg, Mn, Zn, Se, and Cu concentrations (*p* = 0.015, 0.008, 0.014, 0.003, and 0.013, respectively; Additional File S1: Table S3). These overall tests do not establish differences between individual activity groups.

**Figure 1:**
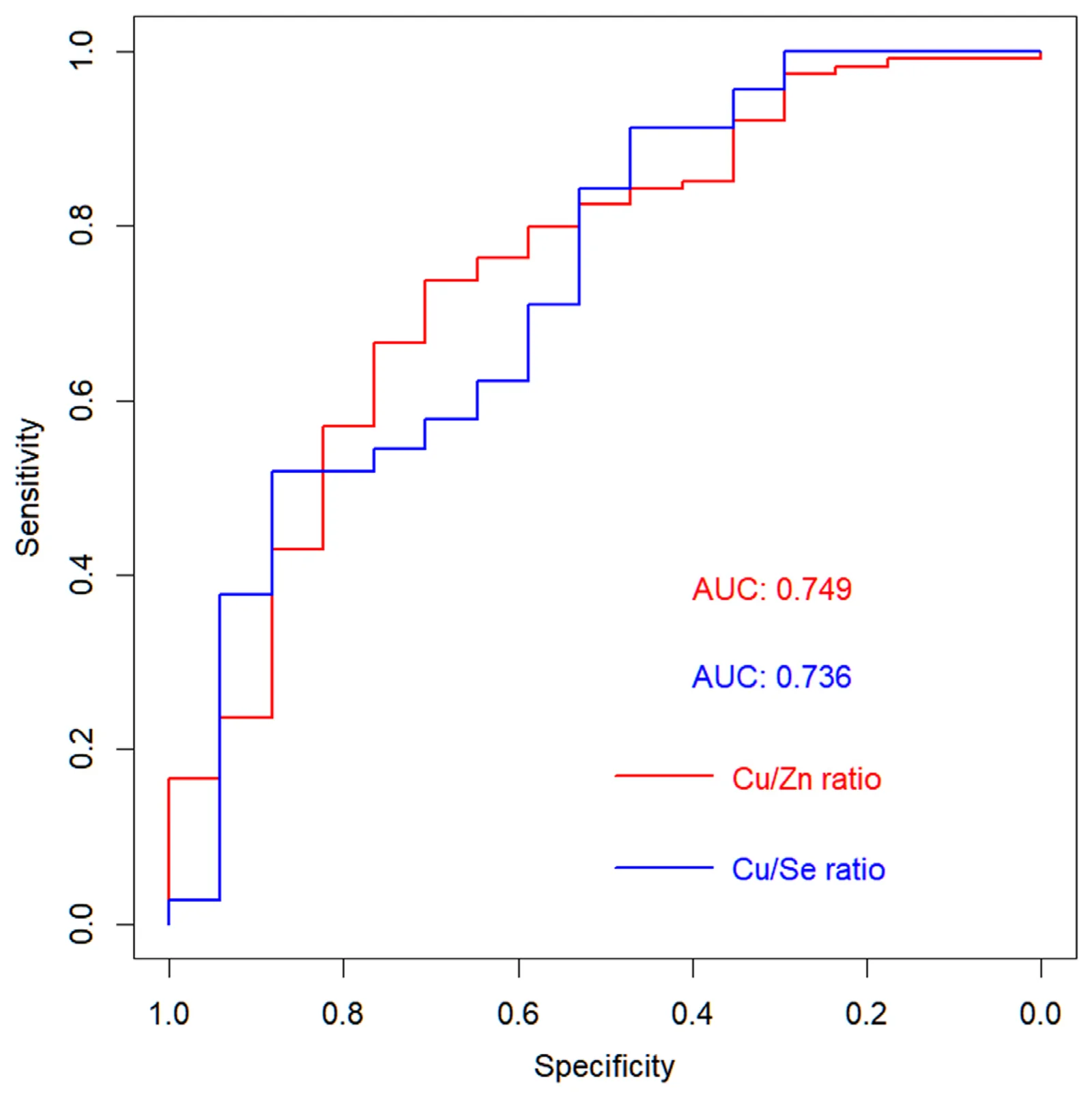
Receiver operating characteristic curves of copper-to-zinc (Cu/Zn) and copper-to-selenium (Cu/Se) ratios for discriminating disease activity. Both ratios demonstrated fair discriminatory ability for remission/mild vs. moderate/severe in patients with inflammatory bowel disease, with area under the curve (AUC) values of 0.749 for the Cu/Zn ratio and 0.736 for the Cu/Se ratio.

**Figure 2:**
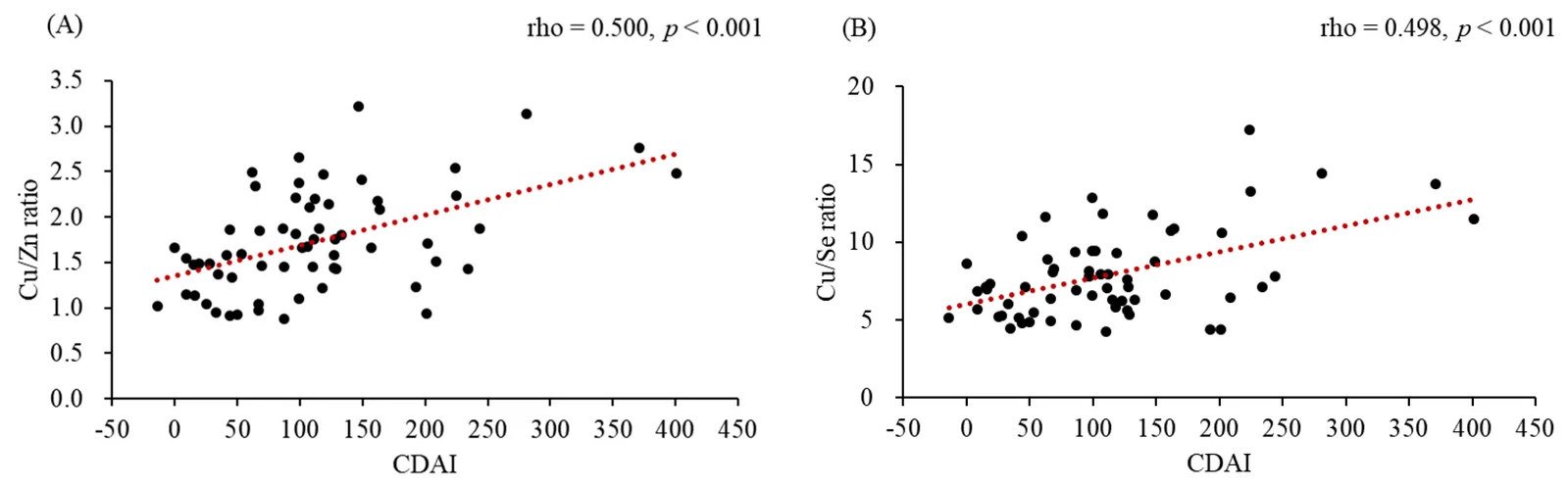
Association of copper-to-zinc (Cu/Zn) and copper-to-selenium (Cu/Se) ratios with clinical disease activity in patient with Crohn’s disease. (**A**) Scatter plot illustrating the positive correlation between the Cu/Zn ratio and Crohn’s Disease Activity Index (CDAI). (**B**) Scatter plot illustrating the positive correlation between the Cu/Se ratio and CDAI.

Serum Cu/Zn and Cu/Se ratios differed across healthy controls and patients with IBD categorized by disease activity, with the highest mean values observed in the moderate-to-severe activity group (Table 2 and Table 3; overall *p* = 0.001 for both ratios). In multivariable linear regression models adjusted for age, sex, and BMI, both ratios were higher in patients with CD than in healthy controls (Cu/Zn: adjusted B = 0.343, 95% CI 0.178–0.509, *p* < 0.001; Cu/Se: adjusted B = 1.671, 95% CI 0.853–2.490, *p* < 0.001). In patients with UC, the Cu/Se ratio was higher than in healthy controls (adjusted B = 0.865, 95% CI 0.075–1.655, *p* = 0.032), whereas the difference in the Cu/Zn ratio was not statistically significant (adjusted B = 0.099, 95% CI −0.061 to 0.258, *p* = 0.222). In separate models evaluating clinical disease activity categories across all patients with IBD, both ratios were higher in the moderate-to-severe activity group than in healthy controls after adjustment for age, sex, and BMI (Cu/Zn: adjusted B = 0.679, 95% CI 0.435–0.923, *p* < 0.001; Cu/Se: adjusted B = 3.857, 95% CI 2.688–5.026, *p* < 0.001). The Cu/Zn ratio was also higher in the mild activity group (*p* = 0.044), whereas the difference between the clinical remission group and healthy controls did not reach statistical significance (*p* = 0.069). The Cu/Se ratio was higher in both the clinical remission and mild activity groups than in healthy controls (*p* = 0.024 and *p* = 0.014, respectively; Additional File S1: Table S6). Receiver operating characteristic curve analysis in patients with IBD yielded areas under the curve of 0.749 and 0.736 for Cu/Zn and Cu/Se, respectively, for distinguishing moderate-to-severe disease from remission or mild disease (Figure 1). In an exploratory analysis of the CD subgroup, the Cu/Zn and Cu/Se ratios yielded AUC values of 0.826 and 0.868, respectively, for distinguishing moderate-to-severe clinical disease activity from remission or mild activity (Supplementary Figure S1). In patients with CD, both ratios were positively correlated with the CDAI (Cu/Zn: rho = 0.500; Cu/Se: rho = 0.498; both *p* < 0.001; Figure 2). In patients with UC, Cu/Zn and Cu/Se ratios were positively correlated with the partial Mayo score (Cu/Zn: rho = 0.363, *p* = 0.002; Cu/Se: rho = 0.277, *p* = 0.021).

### 3.4. Association with Endoscopic Healing

**Figure 3:**
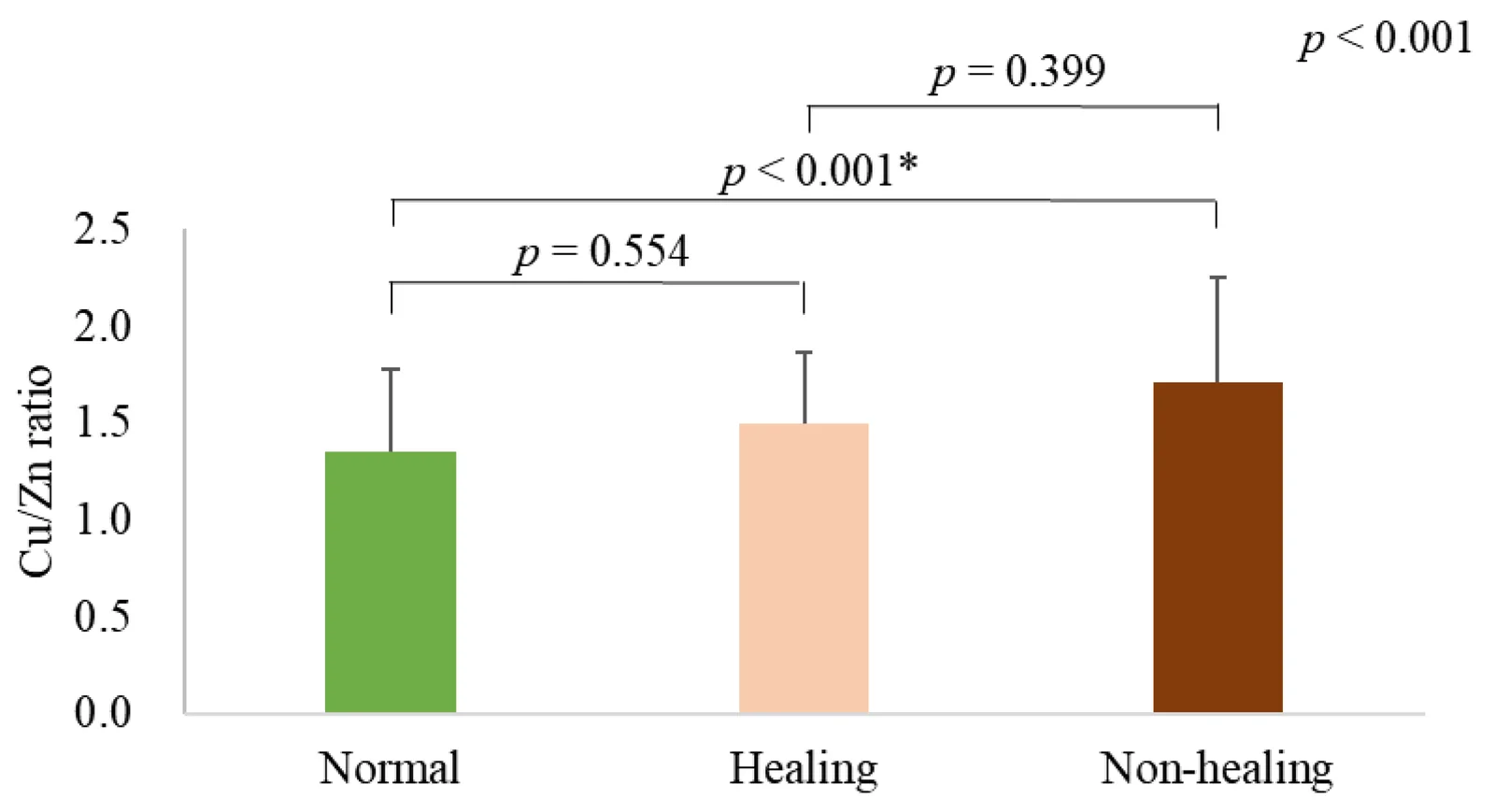
Association of copper-to-zinc (Cu/Zn) ratio with endoscopic healing in patients with inflammatory bowel disease. Comparison of Cu/Zn ratios between patients with and without endoscopic healing. The chart includes *p*-values for pairwise comparisons and overall significance (*p* < 0.001). * Indicates statistical significance.

Serum Mn, Fe, Zn, Se, Mo, and Cu concentrations differed significantly across healthy controls and patients with and without endoscopic healing (overall *p* = 0.004, 0.015, 0.017, 0.020, 0.046, and 0.031, respectively; Additional File S1: Table S4). These overall comparisons do not establish differences between the two patient groups. Cu/Zn ratios also differed across the three groups (overall *p* < 0.001; Table 4 and Figure 3). Although the mean ratio was numerically higher in patients without endoscopic healing than in those with healing (1.711 vs. 1.500), this pairwise difference was not statistically significant (*p* = 0.0399). Similarly, Cu/Se ratios differed across the three groups (overall *p* = 0.003), with mean values of 6.165 in controls, 6.857 in patients with healing, and 7.675 in patients without healing (Additional File S1: Table S5). In analyses stratified by IBD subtype, the overall Cu/Se comparison was significant for CD (*p* = 0.005) but not UC (*p* = 0.053); these findings do not establish a significant healing-versus-non-healing difference within either subtype.

## 4. Discussion

### 4.1. Principal Findings

Serum Cu/Zn and Cu/Se ratios were associated with clinical disease activity in this IBD cohort. Both ratios correlated positively with CDAI in patients with CD and remained higher in CD than in healthy controls after adjustment for age, sex, and BMI. Although the ratios differed significantly across healthy controls and patients with and without endoscopic healing, these overall comparisons do not establish differences between the two patient groups. Baseline characteristics and disease activity distribution may have influenced the findings, and residual confounding by treatment, disease duration, prior bowel resection, and dietary intake remains possible. These results support further investigation of the ratios as potential adjunctive markers but do not establish independent predictive utility.

### 4.2. Interpretation of Trace Element Imbalances

Higher serum Cu and lower Se concentrations were associated with disease activity, consistent with previous reports linking circulating trace element alterations to inflammation [14,15,22,23,24]. These changes may reflect a combination of inflammatory and nutritional influences, although their underlying mechanisms cannot be determined from this study. Altered profiles in patients with limited clinical activity also warrant attention, but lower concentrations relative to controls or elevated ratios do not independently establish nutritional deficiency. Dietary and geographic factors may contribute to variation across cohorts [24]; however, dietary intake was not assessed, and no direct comparison with Western populations was performed. Studies incorporating standardized nutritional assessments and concurrent inflammatory biomarkers are needed to distinguish nutritional depletion from inflammation-associated changes in circulating trace elements.

### 4.3. Study Limitations and Strengths

This study has several limitations. First, the single-center, cross-sectional design and relatively modest sample size preclude definitive conclusions regarding causality between trace element disturbances and disease progression. Without repeated measurements, we could not determine whether Cu/Zn or Cu/Se ratios track changes in disease activity or treatment response. Second, the predominance of patients in clinical remission and the small endoscopic healing subgroup, particularly in CD, limited statistical power and the precision of subgroup estimates. Third, detailed dietary intake data and direct assessments of nutrient absorption or intestinal loss were unavailable, preventing clarification of the mechanisms underlying the observed abnormalities. Fourth, although medication use at enrollment is reported, treatment exposure was not included in the regression models, leaving potential residual confounding. Finally, concurrent fecal calprotectin measurements were unavailable, preventing direct comparisons with this established biomarker and assessment of incremental value. Testing costs and cost-effectiveness were also not evaluated. The ratios should therefore be considered exploratory markers whose clinical role requires validation against established inflammatory biomarkers and standardized nutritional assessments.

Despite these limitations, this study evaluated multiple serum trace elements and their derived ratios in a Taiwanese IBD cohort, incorporating assessments of clinical disease activity and available endoscopic findings. The inclusion of both CD and UC patients and healthy controls allowed comparisons across disease subtypes and clinical activity categories. These data add to the literature on trace element profiles in IBD and provide a basis for further investigation of the observed associations in longitudinal studies. Future prospective multicenter studies incorporating longitudinal follow-up and fecal calprotectin measurements are warranted to validate these findings.

## 5. Conclusions

In this cross-sectional study, serum Cu/Zn and Cu/Se ratios were associated with disease activity in patients with IBD. Both ratios were higher in patients with CD than in healthy controls, and these differences persisted after adjustment for age, sex, and BMI. However, their ability to distinguish patients with and without endoscopic healing and their incremental value beyond established biomarkers remain uncertain. These findings support further investigation of trace element ratios as potential adjunctive markers but do not establish a role for routine clinical monitoring. Multicenter longitudinal studies incorporating fecal calprotectin measurements and standardized nutritional assessments are needed to validate these associations and determine their clinical relevance.

## Figures and Tables

**Table 1:** Clinical characteristics of the enrolled patients (n = 190).

	Total		Normal		CD		UC		
	(n = 190)		(n = 59)		(n = 62)		(n = 69)		*p*-Value
Male, (%)	120	(63.2%)	33	(55.9%)	37	(59.7%)	50	(72.5%)	0.122
Age, mean (years) (SD)	50.93	(15.74)	50.05	(13.88)	52.13	(17.92)	50.61	(15.29)	0.737
BMI, mean (kg/m^2)^ (SD)	23.10	(3.56)	23.85	(3.98)	22.16	(3.64)	23.30	(2.90)	0.011
Disease duration, mean (years) (SD)	7.00	(6.08)			6.89	(6.97)	7.10	(5.20)	0.421
Previous bowel resection, (%)	23	(17.6%)			20	(32.3%)	3	(4.3%)	<0.001
Extraintestinal manifestations, (%)	23	(17.6%)			13	(21.0%)	10	(14.5%)	0.331
Disease activity									0.003
Remission	83	(63.4%)			48	(77.4%)	35	(50.7%)	
Mild	31	(23.7%)			7	(11.3%)	24	(34.8%)	
Moderate/severe	17	(13%)			7	(11.3%)	10	(14.5%)	
UC, Location									
E1: Proctitis							11	(15.9%)	
E2: Left-sided colitis							20	(29.0%)	
E3: Extensive/total colitis							38	(55.1%)	
CD, Location									
L1: Ileal					18	(29.0%)			
L2: Colonic					16	(25.8%)			
L3: Ileocolonic					28	(45.2%)			
CD, Behavior									
B1: Inflammatory					23	(37.1%)			
B2: Stricturing					17	(27.4%)			
B3: Penetrating					22	(35.5%)			
P: Perianal					8	(12.9%)			
Medications									
5-ASA					43	(69.4%)	61	(88.4%)	0.007
Immunosuppressants					33	(53.2%)	24	(34.8%)	0.034
Steroid					23	(37.1%)	15	(21.7%)	0.053
Biologics					16	(25.8%)	6	(8.7%)	0.009

Abbreviations: CD, Crohn’s disease; UC, ulcerative colitis; BMI, body mass index; SD, standard deviation.

**Table 2:** Correlation between serum Cu/Zn ratio and disease activity in patients with IBD.

	IBD			
Healthy individuals (n = 59)	Remission (n = 83)	Mild (n = 31)	Moderate/severe (n = 17)	*p*-value
1.356	1.514	1.490	1.999	0.001
	CD			
Healthy individuals (n = 59)	Remission (n = 48)	Mild (n = 7)	Moderate/severe (n = 7)	*p*-value
1.356	1.649	1.613	2.350	0.001
	UC			
Healthy individuals (n = 59)	Remission (n = 35)	Mild (n = 24)	Moderate/severe (n = 10)	*p*-value
1.356	1.329	1.454	1.753	0.055

Abbreviations: CD, Crohn’s disease; Cu/Zn, copper-to-zinc ratio; IBD, inflammatory bowel disease; UC, ulcerative colitis.

**Table 3:** Correlation between serum Cu/Se ratio and disease activity in patients with IBD.

	IBD			
Healthy individuals (n = 59)	Remission (n = 83)	Mild (n = 31)	Moderate/severe (n = 17)	*p*-value
6.165	6.992	7.022	9.796	0.001
	CD			
Healthy individuals (n = 59)	Remission (n = 48)	Mild (n = 7)	Moderate/severe (n = 7)	*p*-value
6.165	7.280	7.723	12.159	0.001
	UC			
Healthy individuals (n = 59)	Remission (n = 35)	Mild (n = 24)	Moderate/severe (n = 10)	*p*-value
6.165	6.597	6.817	8.141	0.078

Abbreviations: IBD; inflammatory bowel disease; CD, Crohn’s disease; UC; ulcerative colitis; Cu/Se, copper-to-selenium ratio.

**Table 4:** Correlation between serum Cu/Zn ratio and endoscopic healing in patients with IBD.

	IBD		
Healthy individuals (n = 59)	With healing (n = 19)	Without healing (n = 71)	*p*-value
1.356	1.500	1.711	<0.001
	CD		
Healthy individuals (n = 59)	With healing (n = 4)	Without healing (n = 42)	*p*-value
1.356	1.397	1.817	<0.001
	UC		
Healthy individuals (n = 59)	With healing (n = 15)	Without healing (n = 29)	*p*-value
1.356	1.528	1.557	0.038

Abbreviations: IBD; inflammatory bowel disease; CD, Crohn’s disease; UC; ulcerative colitis; Cu/Zn, copper-to-zinc ratio.

## Data Availability

All data generated or analyzed during this study are included in this published article.

## Supplementary Materials

The following supporting information can be downloaded at https://www.mdpi.com/article/10.3390/nu18213593/s1, Table S1: Biochemical data and microelement status of the enrolled patients; Table S2: Correlation between serum microelement levels and disease activity in all patients; Table S3: Correlation between serum microelements and disease activity in patients with CD; Table S4: Correlation between serum microelements and endoscopic healing in patients with IBD; Table S5: Correlation between serum Cu/Se ratio and endoscopic healing in patients; Table S6: Unadjusted and adjusted associations of IBD subtype and disease activity with serum Cu/Zn and Cu/Se ratios; Figure S1: Receiver operating characteristic analysis yielded AUC values for the Cu/Zn and Cu/Se ratios, respectively, for distinguishing moderate-to-severe clinical disease activity from remission or mild activity in the Crohn’s disease cohort.

## Abbreviations

The following abbreviations are used in this manuscript:

CD	Crohn’s disease
CDAI	Crohn’s Disease Activity Index
Cu/Se	Copper-to-selenium ratio
Cu/Zn	Copper-to-zinc ratio
IBD	Inflammatory bowel disease
UC	Ulcerative colitis
