# Supplementary material for: Serum Copper-to-Zinc and Copper-to-Selenium Ratios in Inflammatory Bowel Disease: Possible Associations with Clinical Disease Activity and Endoscopic Healing in a Cross-Sectional Study with Prospective Enrollment
Source: Nutrients. 2026 Oct 29;18(21):3593. doi: 10.3390/nu18213593 (PMC13765236; doi:10.3390/nu18213593)

Additional file S1: Table S1. Biochemical data and microelement status of the enrolled patients

Microelements	Healthy individuals (n = 59)	CD (n = 62)	UC (n = 69)	p-value	p-value (CD vs UC)
Li (μg/L)	1.474	1.577	1.415	0.659	0.364
Mg (μg/L)	21197.494	20195.006	20780.635	**0.013**	**0.036**
Ca (μg/L)	90379.113	87981.933	90194.947	**0.015**	**0.004**
Cr (μg/L)	0.672	0.786	0.691	0.058	0.235
Mn (μg/L)	0.877	0.866	0.827	**0.017**	0.053
Fe (μg/L)	1749.963	1582.506	1441.624	0.050	0.956
Co (μg/L)	0.452	0.545	0.461	0.603	0.588
Ni (μg/L)	0.991	0.760	0.700	0.485	0.510
Cu (μg/L)	909.929	1025.181	961.916	**0.047**	0.139
Zn (μg/L)	709.008	621.378	694.691	**0.001**	**0.001**
Se (μg/L)	153.722	137.044	144.180	**0.009**	0.148
Mo (μg/L)	0.377	0.478	0.427	**0.002**	0.050

Abbreviations: CD, Crohn's disease; UC, ulcerative colitis

Additional file S1: Table S2. Correlation between serum microelement levels and disease activity in all patients

Microelements	Healthy individuals (n = 59)	Remission (n = 83)	Mild (n = 31)	Moderate/severe (n = 17)	p-value
Li (μg/L)	1.474	1.554	1.448	1.266	0.837
Mg (μg/L)	21197.494	20686.52	20176.44	20206.10	0.087
Ca (μg/L)	90379.113	89234.12	90153.03	86891.48	0.420
Cr (μg/L)	0.672	0.740	0.745	0.698	0.196
Mn (μg/L)	0.877	0.878	0.803	0.768	0.106
Fe (μg/L)	1749.963	1589.28	1413.75	1285.36	0.064
Co (μg/L)	0.452	0.482	0.566	0.474	0.637
Ni (μg/L)	0.991	0.685	0.922	0.585	0.375
Cu (μg/L)	909.929	971.16	967.88	1136.63	**0.020**
Zn (μg/L)	709.008	669.08	672.32	593.16	0.055
Se (μg/L)	153.722	143.05	143.63	124.65	**0.004**
Mo (μg/L)	0.377	0.488	0.451	0.269	**0.016**

Additional file S1: Table S3. Correlation between serum microelements and disease activity in patients with CD

Microelements	Healthy individuals (n = 59)	Remission (n = 48)	Mild (n = 7)	Moderate/severe (n = 7)	p-value
Li (µg/L)	1.474	1.594	1.559	1.479	0.923
Mg (µg/L)	21197.494	20380.07	19257.67	19863.32	**0.015**
Ca (µg/L)	90379.113	88364.25	89926.53	83415.71	0.108
Cr (µg/L)	0.672	0.780	0.919	0.696	0.095
Mn (µg/L)	0.877	0.891	0.871	0.693	**0.008**
Fe (µg/L)	1749.963	1671.67	1135.74	1417.86	0.216
Co (µg/L)	0.452	0.501	0.903	0.491	0.485
Ni (µg/L)	0.991	0.739	1.156	0.504	0.167
Cu (µg/L)	909.929	990.46	1032.06	1256.36	**0.013**
Zn (µg/L)	709.008	627.56	651.11	549.27	**0.014**
Se (µg/L)	153.722	140.44	141.80	109.01	**0.003**
Mo (µg/L)	0.377	0.512	0.563	0.162	0.178

Abbreviation: CD, Crohn's disease

Additional file S1: Table S4. Correlation between serum microelements and endoscopic healing in patients with IBD

Microelements	Healthy individuals (n = 59)	IBD with endoscopic healing (n = 19)	IBD without endoscopic healing (n = 71)	p-value
Li (µg/L)	1.474	1.331	1.501	0.985
Mg (µg/L)	21197.494	20694.89	20408.08	0.069
Ca (µg/L)	90379.113	90304.73	88277.98	0.116
Cr (µg/L)	0.672	0.714	0.736	0.198
Mn (µg/L)	0.877	0.695	0.780	**0.004**
Fe (µg/L)	1749.963	1349.07	1389.87	**0.015**
Co (µg/L)	0.452	0.461	0.514	0.845
Ni (µg/L)	0.991	0.641	0.758	0.492
Cu (µg/L)	909.929	972.87	1023.53	**0.031**
Zn (µg/L)	709.008	666.76	624.55	**0.017**

Se (μg/L)	153.722	148.21	139.75	**0.020**
Mo (μg/L)	0.377	0.429	0.464	**0.046**

Abbreviation: IBD; inflammatory bowel disease

Additional file S1: Table S5. Correlation between serum Cu/Se ratio and endoscopic healing in patients.

	IBD		
Healthy individuals (n = 59)	With healing (n = 19)	Without healing (n = 71)	p-value
6.165	6.857	7.675	**0.003**

	CD		
Healthy individuals (n = 59)	With healing (n = 4)	Without healing (n = 42)	p-value
6.165	6.649	7.935	**0.005**

	UC		
Healthy individuals (n = 59)	With healing (n = 15)	Without healing (n = 29)	p-value
6.165	6.912	7.298	0.053

Abbreviations: IBD; inflammatory bowel disease; CD, Crohn’s disease; UC; ulcerative colitis

Additional file S1: Table S6. Unadjusted and adjusted associations of IBD subtype and disease activity with serum Cu/Zn and Cu/Se ratios

A. Cu/Zn ratio

Group	Mean ± SD	Unadjusted B (95% CI)	p-value	Adjusted B (95% CI)	p-value
**IBD subtype**					
Healthy controls	1.36 ± 0.42	Reference	—	Reference	—
CD	1.72 ± 0.56	0.369 (0.201 to 0.537)	**<0.001**	0.343 (0.178 to 0.509)	**<0.001**
UC	1.43 ± 0.41	0.078 (-0.086 to 0.242)	0.348	0.099 (-0.061 to 0.258)	0.222
**Disease activity**					
Healthy controls	1.36 ± 0.42	Reference	—	Reference	—
Remission	1.51 ± 0.47	0.158 (0.002 to 0.315)	**0.047**	0.137 (-0.011 to 0.285)	0.069
Mild	1.49 ± 0.41	0.134 (-0.069 to 0.338)	0.194	0.204 (0.005 to 0.402)	**0.044**
Moderate/severe	2.00 ± 0.63	0.643 (0.391 to 0.896)	**<0.001**	0.679 (0.435 to 0.923)	**<0.001**

B. Cu/Se ratio

Group	Mean ± SD	Unadjusted B (95% CI)	p-value	Adjusted B (95% CI)	p-value
**IBD subtype**					
Healthy controls	6.16 ± 1.98	Reference	—	Reference	—
CD	7.88 ± 2.82	1.716 (0.904 to 2.528)	**<0.001**	1.671 (0.853 to 2.490)	**<0.001**
UC	6.90 ± 1.89	0.733 (-0.059 to 1.524)	0.069	0.865 (0.075 to 1.655)	**0.032**
**Disease activity**					
Healthy controls	6.16 ± 1.98	Reference	—	Reference	—
Remission	6.99 ± 1.95	0.827 (0.099 to 1.555)	**0.026**	0.817 (0.108 to 1.527)	**0.024**
Mild	7.02 ± 1.97	0.857 (-0.092 to 1.805)	0.076	1.200 (0.250 to 2.150)	**0.014**
Moderate/severe	9.80 ± 3.69	3.631 (2.454 to 4.808)	**<0.001**	3.857 (2.688 to 5.026)	**<0.001**

Values are presented as mean ± SD. B denotes the unstandardized regression coefficient; 95% CIs are shown in parentheses. Healthy controls are the reference group. Adjusted models include age, sex, and BMI. Bold p-values indicate $p < 0.05$.

Abbreviations: IBD, inflammatory bowel disease; CD, Crohn's disease; UC, ulcerative colitis; Cu, copper; Zn, zinc; Se, selenium; SD, standard deviation; CI, confidence interval; BMI, body mass index.

Additional file S1: Figure S1. Receiver operating characteristic analysis yielded AUC values for the Cu/Zn and Cu/Se ratios, respectively, for distinguishing moderate-to-severe clinical disease activity from remission or mild activity in the Crohn's disease cohort

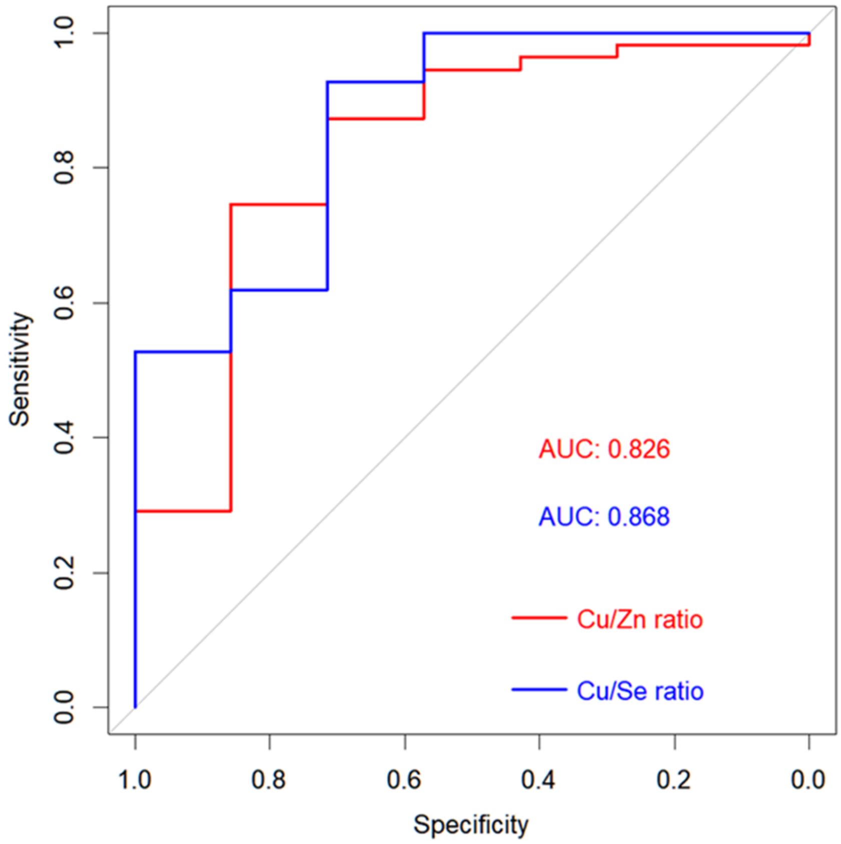

Supplement: Supplementary file 1 [file nutrients-18-03593-s001.zip › nutrients-4541746-supplementary.pdf]
